# A nomogram based on the red cell distribution width to lymphocyte ratio as a prognostic tool for non-muscle-invasive bladder cancer: a retrospective study

**DOI:** 10.3389/fonc.2026.1728821

**Published:** 2026-01-29

**Authors:** Feifan Song, Shiqiang Su, Xueqiao Zhang, Yunpeng Cao, Xiongjie Cui, Lili Zhang, Chao Li, Shen Li, Shuo Tian, Lizhe Liu

**Affiliations:** 1 Department of Urology, Shijiazhuang People’s Hospital, Shijiazhuang, China; 2 Graduate School of Hebei Medical University, Shijiazhuang, China; 3 Institute of Medicine and Health, Hebei Medical University, Shijiazhuang, China

**Keywords:** lymphocyte count, nomogram, non-muscle-invasive bladder cancer, prognosis, red cell distribution width

## Abstract

**Background:**

This study investigated the prognostic value of the preoperative red cell distribution width to lymphocyte ratio (RLR) for recurrence-free survival (RFS) and overall survival (OS) in patients with non-muscle-invasive bladder cancer (NMIBC) undergoing transurethral resection of bladder tumor (TURBT).

**Methods:**

A retrospective analysis was performed on data from 239 patients who received TURBT. The optimal RLR cutoff was determined using time-dependent receiver operating characteristic (ROC) curve analysis. Survival outcomes were assessed using Kaplan-Meier curves and univariate/multivariate Cox regression. An RFS prognostic nomogram incorporating independent factors was developed and evaluated via the concordance index (C-index), calibration plots, time-dependent ROC, and decision curve analysis (DCA). Subgroup analyses assessed the consistency of the RLR-RFS association.

Results: Elevated preoperative RLR was an independent prognostic factor for worse RFS and OS. Moreover, compared to the platelet-to-lymphocyte ratio (PLR) and neutrophil-to-lymphocyte ratio (NLR), RLR demonstrated relatively higher predictive performance for 1-year and 3-year RFS. The novel nomogram incorporating the RLR parameter demonstrated improved predictive accuracy and a trend toward greater net clinical benefit compared to the conventional model based solely on the EORTC recurrence risk classification. Subgroup analysis indicated a stronger relationship between high RLR and poor RFS in patients with pTaN0M0 compared to those with pT1N0M0.

**Conclusion:**

Preoperative RLR represents a potential independent prognostic indicator for tumor recurrence in NMIBC patients following TURBT. The RLR-based nomogram may improve recurrence risk prediction in this population.

## Introduction

As a predominant malignancy of the urinary tract, bladder cancer is expected to account for approximately 84,870 new cases and 17,420 deaths in the United States in 2025 (1). Clinically, non-muscle-invasive bladder cancer (NMIBC) accounts for approximately 70% of bladder cancer cases at initial presentation. Although NMIBC has a relatively favorable prognosis, its high recurrence rate necessitates long-term and frequent cystoscopic surveillance for patients. This not only imposes a substantial healthcare economic burden but also significantly impacts patients’ physical and psychological well-being (2). Consequently, to address the challenge of optimizing management strategies for NMIBC, there is a critical need for prognostic tools that can precisely stratify the risk of recurrence.

Stratification of recurrence risk in NMIBC is currently guided by conventional clinicopathological characteristics, such as tumor grade and stage, as well as size, multiplicity, and the status of CIS (carcinoma in situ). The EORTC (European Organization of Research and Treatment of Cancer) risk scoring system, recommended by the EAU (European Association of Urology), is a classic tool based on these factors (3). However, such models have limitations, and their predictive accuracy requires further improvement. A growing research focus has been placed on the roles of the tumor microenvironment and systemic inflammatory response in tumorigenesis, progression, and prognosis. Inflammatory responses contribute to tumor initiation and proliferation and are key drivers of angiogenesis, invasion, and metastatic spread (4). Notably, immune cell infiltration, inflammatory cytokine networks, and their regulatory signaling pathways have emerged as an important immunological basis influencing tumor behavior (5, 6). Consequently, exploring easily accessible and cost-effective immune-related biomarkers that can effectively reflect the body’s inflammatory status, and integrating them into existing prognostic evaluation systems, holds significant clinical value for enhancing risk assessment and inform personalized treatment decisions.

The red cell distribution width to lymphocyte ratio (RLR) is defined as an integrative biomarker representing aspects of both inflammation and immune response. Red cell distribution width (RDW), an indicator of red blood cell volume variation, is influenced not only by erythropoiesis but also regulated by inflammatory cytokines such as interleukin-6, thereby serving as a sensitive indicator of systemic inflammatory status (7). There is growing evidence that an elevated RDW correlates independently with poorer outcomes in a spectrum of diseases, such as multiple cancers and cardiovascular conditions (8, 9). Conversely, the lymphocyte count directly reflects the body’s anti-tumor immune capacity (10). By integrating these two parameters, RLR theoretically provides a more comprehensive reflection of the balance between systemic inflammation and immune status. Emerging evidence supports the prognostic utility of RLR in several cancer types, such as renal cell carcinoma (11), cutaneous melanoma (12), and upper tract urothelial carcinoma ​ (13). Nevertheless, the prognostic significance of RLR specifically in patients with NMIBC remains unexplored.

To address the need for better prognostic tools in NMIBC, this study sought to determine whether preoperative RLR predicts recurrence-free survival (RFS) and overall survival (OS) in patients undergoing TURBT, thereby appraising its clinical applicability as a novel biomarker.

## Materials and methods

### Study population

**Figure 1:**
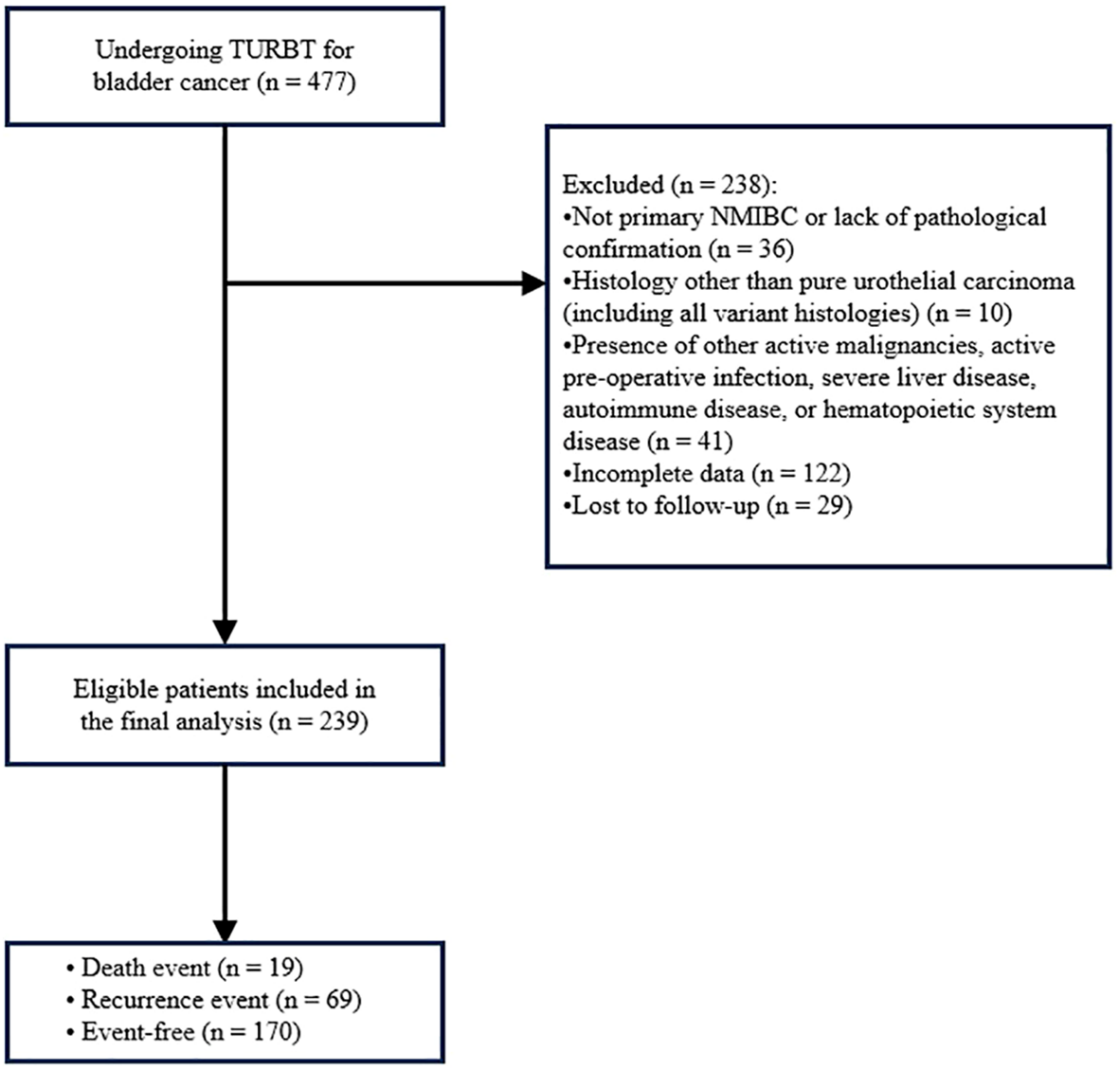
The flow chart for patient selection.

Patients with NMIBC undergoing TURBT from November 2013 to January 2024 at Shijiazhuang People’s Hospital were identified for this retrospective analysis. Eligible patients met the following criteria: 1) initial diagnosis of bladder urothelial carcinoma confirmed by postoperative pathological examination, meeting the diagnostic criteria for NMIBC; and 2) availability of complete data on RDW, lymphocyte count, other clinicopathological characteristics, and follow-up information. Cases were excluded based on: 1) history of other malignancies, or presence of lymph node metastasis or distant metastasis at initial diagnosis (i.e., muscle-invasive bladder cancer or advanced bladder cancer); 2) non-primary NMIBC or lack of pathological confirmation, and non-pure urothelial carcinoma histology (including all variant histologies). 3) pre-existing autoimmune diseases, active acute or chronic infections, or known hematological disorders; 4) perioperative blood transfusion, or history of major trauma or surgery within one month prior to the TURBT procedure. Ultimately, 239 patients were enrolled in this study. Among them, 19 patients died, 69 experienced recurrence, and 170 remained event-free (**Figure 1**). Prior approval for this study was obtained from the Ethics Committee of Shijiazhuang People’s Hospital, and all methods were conducted in compliance with the Declaration of Helsinki.

### Variables

Demographic characteristics, hematological parameters, and information regarding postoperative instillation therapy were extracted from the digital medical records. Demographic characteristics included gender, age, smoking history, history of previous abdominal surgery, and history of common chronic diseases. Hematological parameters comprised the platelet-to-lymphocyte ratio (PLR), neutrophil-to-lymphocyte ratio (NLR), RLR. Fasting venous blood samples were obtained within the week preceding surgery, and all hematological parameters were measured from these samples. The hospital’s central laboratory conducted all hematological measurements following standardized protocols on automated analytical platforms. Tumor pathological characteristics included tumor grade, stage, tumor size, multiplicity and concomitant CIS. Tumor grading was assessed using both the 1973 and 2004/2016 World Health Organization (WHO) classifications, while tumor staging adhered to the American Joint Committee on Cancer TNM system (8th edition, 2017). Furthermore, patients in this study cohort were stratified into different risk groups based on the total score of the six clinicopathological factors associated with recurrence according to the EORTC scoring system (14), and were also classified into different risk categories based on the prognostic factor risk stratification outlined in the EAU guidelines (15).

### ​​Follow-up and outcomes

​​All enrolled patients were followed up according to a standard protocol. We performed standardized follow-up for all included patients. Specifically, the follow-up schedule was structured as follows: assessments at 3-month intervals for the initial 2 years after surgery, extending to 6-month intervals for the next 3 years, and yearly after that. Evaluations comprised urine analysis, urinary cytology, urinary tract ultrasonography, and cystoscopy. The main outcome, RFS, was measured as the time span from TURBT to the initial confirmed event (intravesical recurrence or distant metastasis). The secondary outcome was OS, calculated from the surgical date until mortality due to any reason.

### Statistical analysis

Time-dependent ROC curves were employed to determine the optimal cutoff values for RLR, NLR, and PLR in predicting 1-year RFS in NMIBC. Subsequently, the cohort was dichotomized into high and low RLR groups based on the established optimal cutoff value. Continuous data are presented as medians along with their interquartile ranges, with group comparisons performed using the Mann-Whitney U test. For categorical data, which are reported as frequencies (percentages), the Chi-square test or Fisher’s exact test was applied as appropriate. Survival probabilities were estimated with the Kaplan-Meier method, and the log-rank test was employed to evaluate differences between the groups. Prognostic factors influencing RFS and OS were screened using univariate and multivariate Cox regression analyses. The association strength of identified factors was quantified by hazard ratios (HR) and corresponding 95% confidence intervals (CI). To construct a predictive nomogram for 1-, 3-, and 5-year RFS, we selected all variables that remained significant (P < 0.05) in the multivariate analysis, designating them as independent prognostic factors. The discriminatory ability of the nomogram was assessed by the concordance index (C-index) and time-dependent ROC curves. Calibration curves, along with bootstrap resampling (1000 repetitions), were used for internal validation to assess the nomogram’s calibration accuracy and stability. Decision curve analysis (DCA) was further conducted to estimate the clinical utility of the nomogram across different threshold probabilities. Subgroup analyses for RFS were performed to evaluate the consistency of the association between RLR and survival across various patient characteristics, with P-values for interaction calculated to assess effect heterogeneity. The analyses were done in R (version 4.4.3). Two-sided statistical tests were used, and a P-value < 0.05 was deemed to be significant throughout the study.

## Results

### Patient features

**Figure 2:**
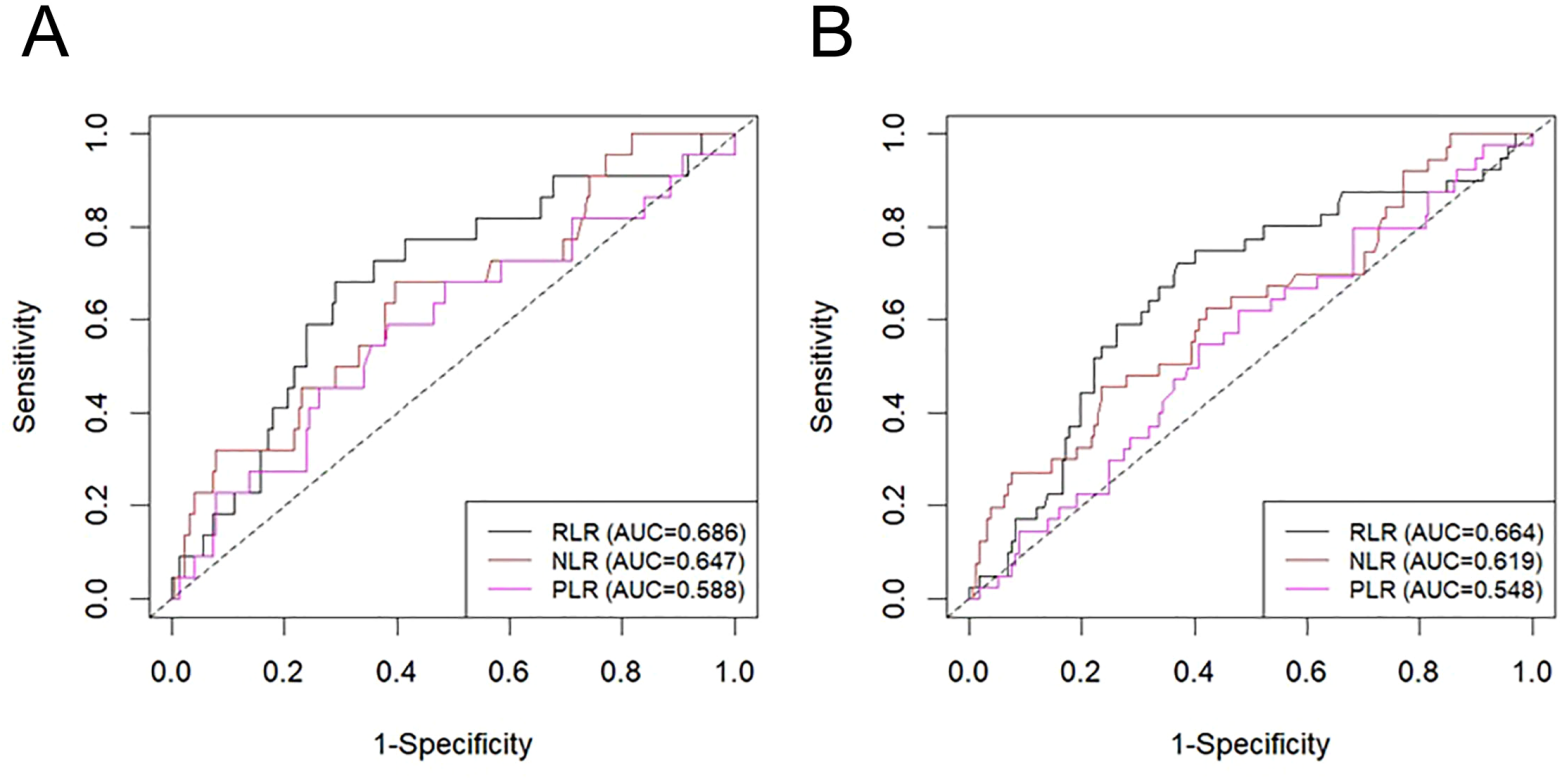
Time-dependent ROC curves for the prediction of 1-year RFS **(A)** and 3-year RFS **(B)**. RFS, recurrence-free survival; RLR, red blood cell distribution width-to-lymphocyte ratio; NLR, neutrophil-to-lymphocyte ratio; PLR, platelet-to-lymphocyte ratio; AUC: area under the curve.

This final analysis included 239 patients with NMIBC. The cohort had a median age of 69 years (interquartile ranges: 60-77), with 25 females (10.46%) and 214 males (89.54%). The median follow-up time was 58.15 months. The optimal cutoff values, defined by time-dependent ROC analysis (**Figure 2A**), were 6.85 for the RLR, 2.05 for the NLR, and 111.95 for the PLR (**Supplementary Table S1**). Two cohorts were established based on the RLR cutoff (6.85): a low-RLR group (n= 82) and a high-RLR group (n=157). The baseline clinical characteristics of the two groups are detailed in **Table 1**. Comparative analysis demonstrated no significant differences between the two groups in terms of gender, age, smoking history, diabetes, coronary heart disease, history of abdominal surgery, tumor number, tumor size, tumor grade (WHO1973), Tumor grade (WHO 2004/2016), tumor stage, concomitant CIS, postoperative instillation therapy, EORTC recurrence risk classification, EAU risk stratification, NLR, or PLR.

**Table 1:** Study characteristics.

Characteristic	Overall N = 239	Low RLR N = 82	High RLR N = 157	P value
Gender				0.527
Female	25 (10.46%)	10 (12.20%)	15 (9.55%)	
Male	214 (89.54%)	72 (87.80%)	142 (90.45%)	
Age, years	69.00 (60.00, 77.00)	68.00 (60.00, 77.00)	69.00 (60.00, 77.00)	0.735
Smoking				0.104
No	162 (67.78%)	50 (60.98%)	112 (71.34%)	
Yes	77 (32.22%)	32 (39.02%)	45 (28.66%)	
Diabetes				0.362
No	205 (85.77%)	68 (82.93%)	137 (87.26%)	
Yes	34 (14.23%)	14 (17.07%)	20 (12.74%)	
Coronary heart disease				0.127
No	209 (87.45%)	68 (82.93%)	141 (89.81%)	
Yes	30 (12.55%)	14 (17.07%)	16 (10.19%)	
History of abdominal surgery				0.603
No	205 (85.77%)	69 (84.15%)	136 (86.62%)	
Yes	34 (14.23%)	13 (15.85%)	21 (13.38%)	
Number of tumors				0.534
Single	155 (64.85%)	51 (62.20%)	104 (66.24%)	
Multiple	84 (35.15%)	31 (37.80%)	53 (33.76%)	
Tumor size, cm	2.50 (2.00, 3.00)	3.00 (2.00, 3.00)	2.50 (2.00, 3.00)	0.311
Tumor grade (WHO1973)				0.963
G1	137 (57.32%)	48 (58.54%)	89 (56.69%)	
G2	30 (12.55%)	10 (12.20%)	20 (12.74%)	
G3	72 (30.13%)	24 (29.27%)	48 (30.57%)	
Tumor grade (WHO 2004/2016)				0.234
Low grade	139 (58.16%)	52 (63.41%)	87 (55.41%)	
High grade	100 (41.84%)	30 (36.59%)	70 (44.59%)	
Tumor stage				0.403
pTaN0M0	131 (54.81%)	48 (58.54%)	83 (52.87%)	
pT1N0M0	108 (45.19%)	34 (41.46%)	74 (47.13%)	
Concomitant CIS				0.667
No	233 (97.49%)	81 (98.78%)	152 (96.82%)	
Yes	6 (2.51%)	1 (1.22%)	5 (3.18%)	
Instillation therapy				0.189
Immunotherapy	33 (13.81%)	8 (9.76%)	25 (15.92%)	
Chemotherapy	206 (86.19%)	74 (90.24%)	132 (84.08%)	
EORTC recurrence risk classification				0.320
0 (Low risk)	27 (11.30%)	12 (14.63%)	15 (9.55%)	
1–4 (Intermediate low risk)	121 (50.63%)	36 (43.90%)	85 (54.14%)	
5-9(Intermediate high risk)	90 (37.66%)	34 (41.46%)	56 (35.67%)	
10-17(High risk)	1 (0.42%)	0 (0.00%)	1 (0.64%)	
EAU risk stratification				0.332
Low risk	84 (35.15%)	35 (42.68%)	49 (31.21%)	
Intermediate risk	88 (36.82%)	28 (34.15%)	60 (38.22%)	
High risk	55 (23.01%)	15 (18.29%)	40 (25.48%)	
Very high risk	12 (5.02%)	4 (4.88%)	8 (5.10%)	
NLR				0.368
≤2.05	107 (44.77%)	40 (48.78%)	67 (42.68%)	
>2.05	132 (55.23%)	42 (51.22%)	90 (57.32%)	
PLR				0.128
≤111.95	92 (38.49%)	37 (45.12%)	55 (35.03%)	
>111.95	147 (61.51%)	45 (54.88%)	102 (64.97%)	
Follow-up duration, months	58.15 (36.14, 98.78)	71.91 (40.08, 105.35)	57.39 (35.25, 96.75)	0.237

RLR, red blood cell distribution width-to-lymphocyte ratio; CIS, Carcinoma in situ; EORTC, European Organization of Research and Treatment of Cancer; EAU, European Association of Urology; NLR, neutrophil-to-lymphocyte ratio; PLR, platelet-to-lymphocyte ratio.

Continuous variables are expressed as medians (interquartile range), while categorical variables are expressed as sample sizes (percentages).

### Association between RLR and RFS/OS

**Figure 3:**
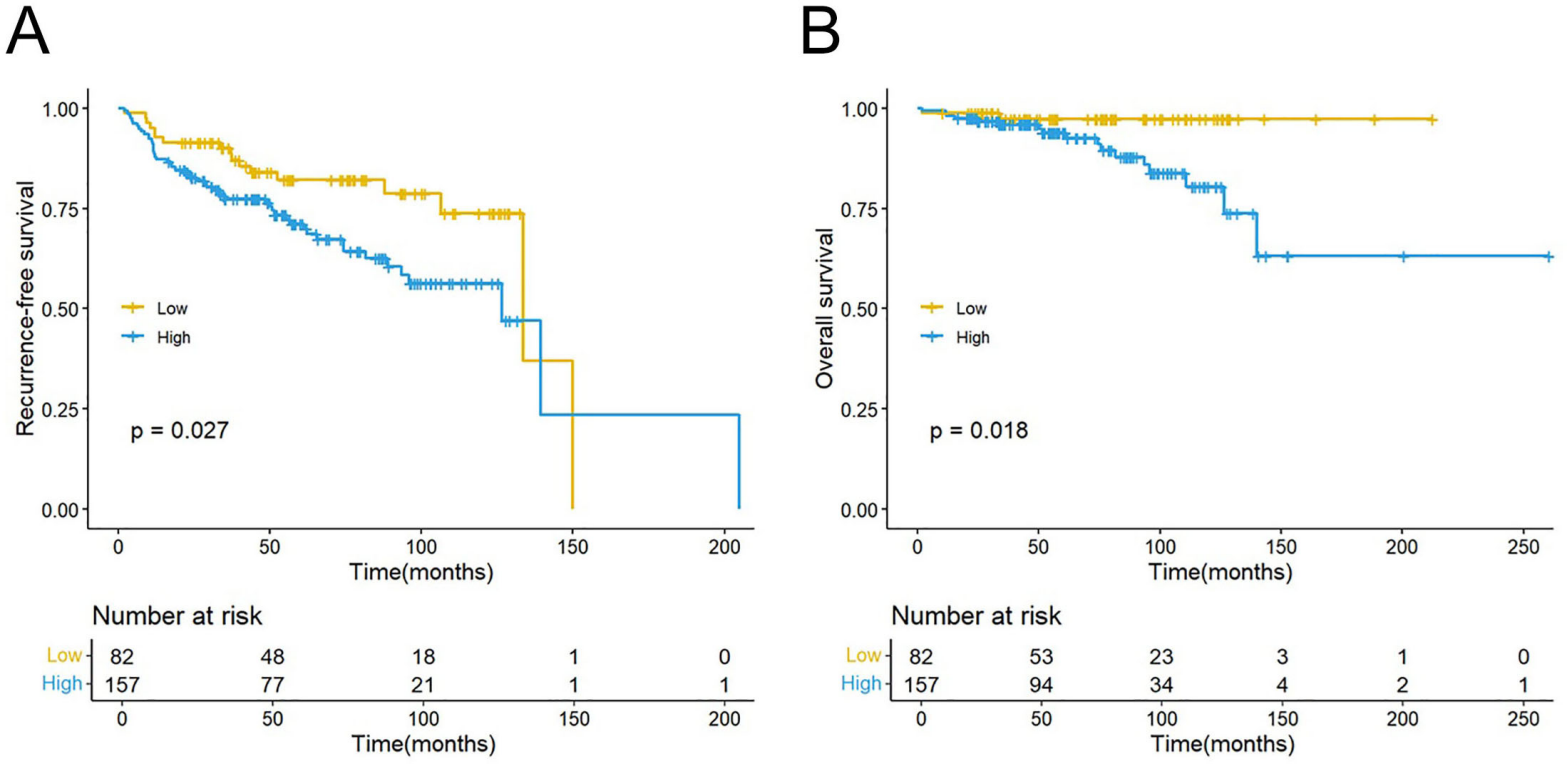
Kaplan–Meier curves for RFS **(A)** and OS **(B)** stratified by the RLR. RFS, recurrence-free survival; OS, overall survival; RLR, red blood cell distribution width-to-lymphocyte ratio.

A significant reduction in both RFS (P = 0.027) and OS (P = 0.018) was demonstrated in the high-RLR group relative to the low-RLR group by Kaplan-Meier analysis, as illustrated in **Figures 3A, B**, respectively. Univariate Cox regression analysis revealed a high RLR as a significant predictor for both shorter RFS (HR: 1.847, 95%CI: 1.065–3.202; P = 0.029; **Table 2**) and poorer OS (HR: 4.93, 95% CI: 1.138–21.361); P = 0.033; **Table 3**). Following adjustment for possible confounders, a multivariate Cox regression was conducted. A high preoperative RLR remained an independent predictor for both RFS (HR: 2.113, 95% CI: 1.207–3.700, P = 0.009; **Table 2**) and OS (HR: 4.989, 95% CI: 1.102–22.591, P = 0.037; **Table 3**). The comparative the predictive ability of RLR against NLR and PLR in predicting RFS was evaluated using time-dependent ROC curves. In predicting 1-year RFS, the AUC of RLR (0.686) was numerically higher​ than that of NLR (0.647) and PLR (0.588) (**Figure 2A**). Similarly, for 3-year RFS prediction, RLR also demonstrated a higher AUC (0.664) compared to NLR (0.619) and PLR (0.548) (**Figure 2B**).

**Table 2:** Univariate and multivariate cox regression analysis for recurrence-free survival.

Characteristic	Univariate analysis		Multivariate analysis	
	HR (95% CI)	P value	HR (95% CI)	P value
Gender				
Female	Reference			
Male	2.593 (0.813–8.266)	0.107		
Age, years	1.030 (1.008–1.052)	0.008	1.023 (1.000–1.046)	0.049
Smoking				
No	Reference			
Yes	0.961 (0.580–1.594)	0.878		
Diabetes				
No	Reference			
Yes	0.899 (0.445–1.818)	0.767		
Coronary heart disease				
No	Reference			
Yes	1.253 (0.638–2.461)	0.513		
History of abdominal surgery				
No	Reference		Reference	
Yes	2.192 (1.188–4.047)	0.012	2.664 (1.417–5.010)	0.002
Tumor number				
Single	Reference			
Multiple	0.839 (0.504–1.395)	0.498		
Tumor size, cm	1.506 (1.261–1.798)	<0.001	1.519 (1.280–1.803)	<0.001
Tumor grade (WHO1973)				
G1	Reference			
G2	0.841 (0.387–1.825)	0.661		
G3	0.975 (0.574–1.656)	0.926		
Tumor grade (WHO 2004/2016)				
Low grade	Reference			
High grade	1.113 (0.690–1.795)	0.66		
Tumor stage				
pTaN0M0	Reference		Reference	
pT1N0M0	1.972 (1.197–3.247)	0.008	2.140 (1.288–3.557)	0.003
Concomitant CIS				
No	Reference		Reference	
Yes	5.060 (2.177–11.760)	<0.001	6.377 (2.650–15.349)	<0.001
Instillation therapy				
Immunotherapy	Reference			
Chemotherapy	0.565 (0.317–1.007)	0.053		
NLR				
≤2.05	Reference			
>2.05	1.520 (0.932–2.479)	0.094		
PLR				
≤111.95	Reference			
>111.95	1.204 (0.733–1.978)	0.463		
RLR				
≤6.85	Reference		Reference	
>6.85	1.847 (1.065–3.202)	0.029	2.113 (1.207–3.700)	0.009

HR, hazard ratio; CI, confidence interval; CIS, Carcinoma in situ; NLR, neutrophil-to-lymphocyte ratio; PLR, platelet-to-lymphocyte ratio; RLR, red blood cell distribution width-to-lymphocyte ratio.

**Table 3:** Univariate and multivariate cox regression analysis for overall survival.

Characteristic	Univariate analysis		Multivariate analysis	
	HR (95% CI)	P value	HR (95% CI)	P value
Gender				
Female	Reference			
Male	1.96 (0.260–14.808)	0.513		
Age, years	1.09 (1.031–1.142)	0.002	1.066 (1.009–1.127)	0.024
Smoking				
No	Reference			
Yes	0.494 (0.164–1.491)	0.211		
Diabetes				
No	Reference			
Yes	1.53 (0.503–4.634)	0.456		
Coronary heart disease				
No	Reference			
Yes	1.09 (0.315–3.789)	0.889		
History of abdominal surgery				
No	Reference			
Yes	0.458 (0.061–3.462)	0.449		
Tumor number				
Single	Reference			
Multiple	0.751 (0.268–2.104)	0.585		
Tumor size, cm	1.97 (1.468–2.630)	<0.001	1.770 (1.323–2.369)	<0.001
Tumor grade (WHO1973)				
G1	Reference			
G2	1.03 (0.222–4.801)	0.967		
G3	1.51 (0.582–3.926)	0.396		
Tumor grade (WHO 2004/2016)				
Low grade	Reference			
High grade	1.43 (0.581–3.534)	0.434		
Tumor stage				
pTaN0M0	Reference			
pT1N0M0	2.46 (0.875–6.931)	0.088		
Concomitant CIS				
No	Reference			
Yes	3.43 (0.782–15.056)	0.102		
Instillation therapy				
Immunotherapy	Reference		Reference	
Chemotherapy	0.314 (0.123–0.801)	0.015	0.572 (0.215–1.517)	0.262
NLR				
≤2.05	Reference			
>2.05	1.40 (0.559–3.497)	0.473		
PLR				
≤111.95	Reference			
>111.95	1.07 (0.430–2.679)	0.879		
RLR				
≤6.85	Reference		Reference	
>6.85	4.93 (1.138–21.361)	0.033	4.989 (1.102–22.591)	0.037

HR, hazard ratio; CI, confidence interval; CIS, Carcinoma in situ; NLR, neutrophil-to-lymphocyte ratio; PLR, platelet-to-lymphocyte ratio; RLR, red blood cell distribution width-to-lymphocyte ratio.

### ​​Nomogram construction and performance​​

**Figure 4:**
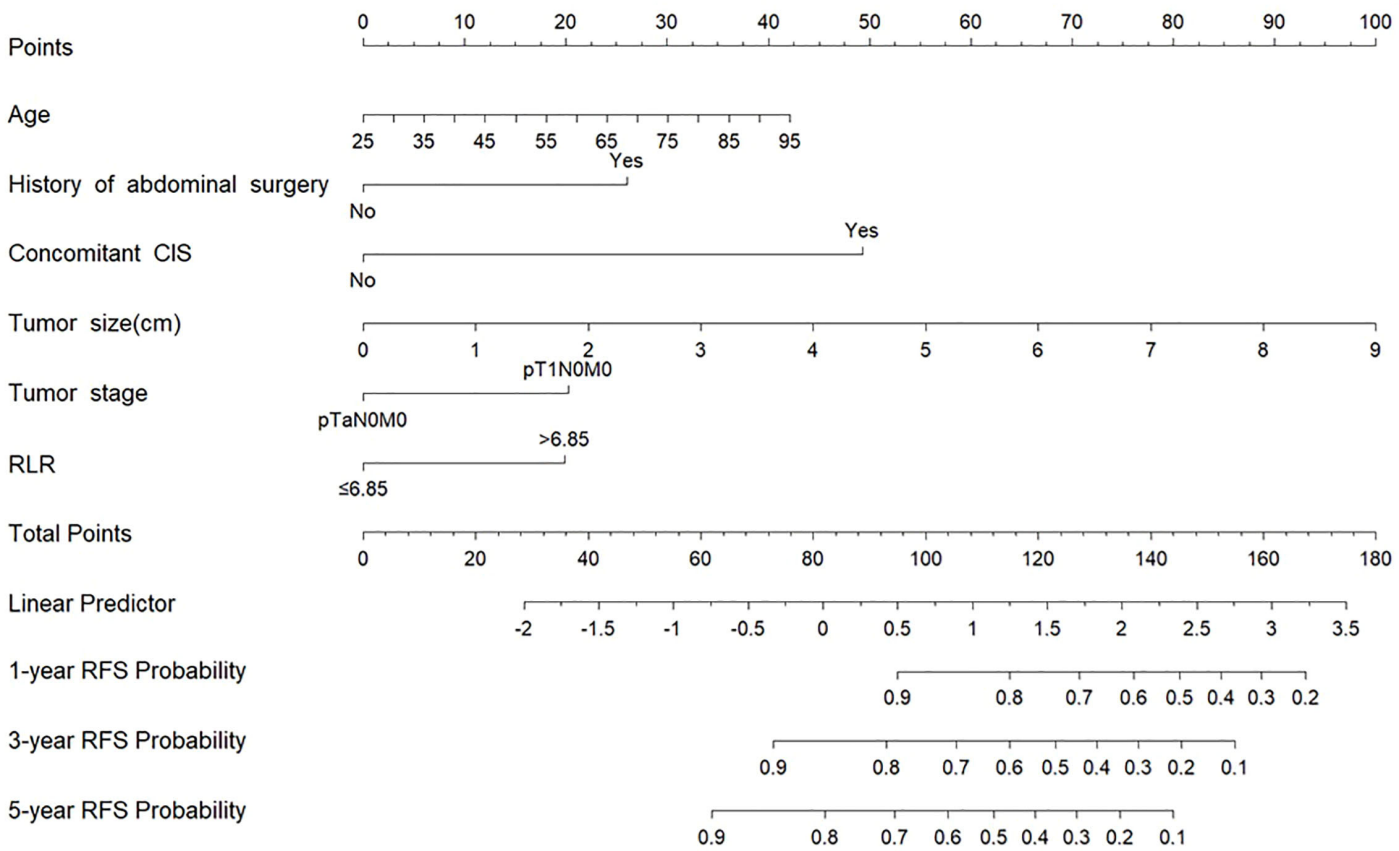
Nomograms for the prediction of 1-, 3- and 5-year RFS. RFS, recurrence-free survival; CIS, Carcinoma in situ; RLR, red blood cell distribution width-to-lymphocyte ratio.

**Figure 5:**
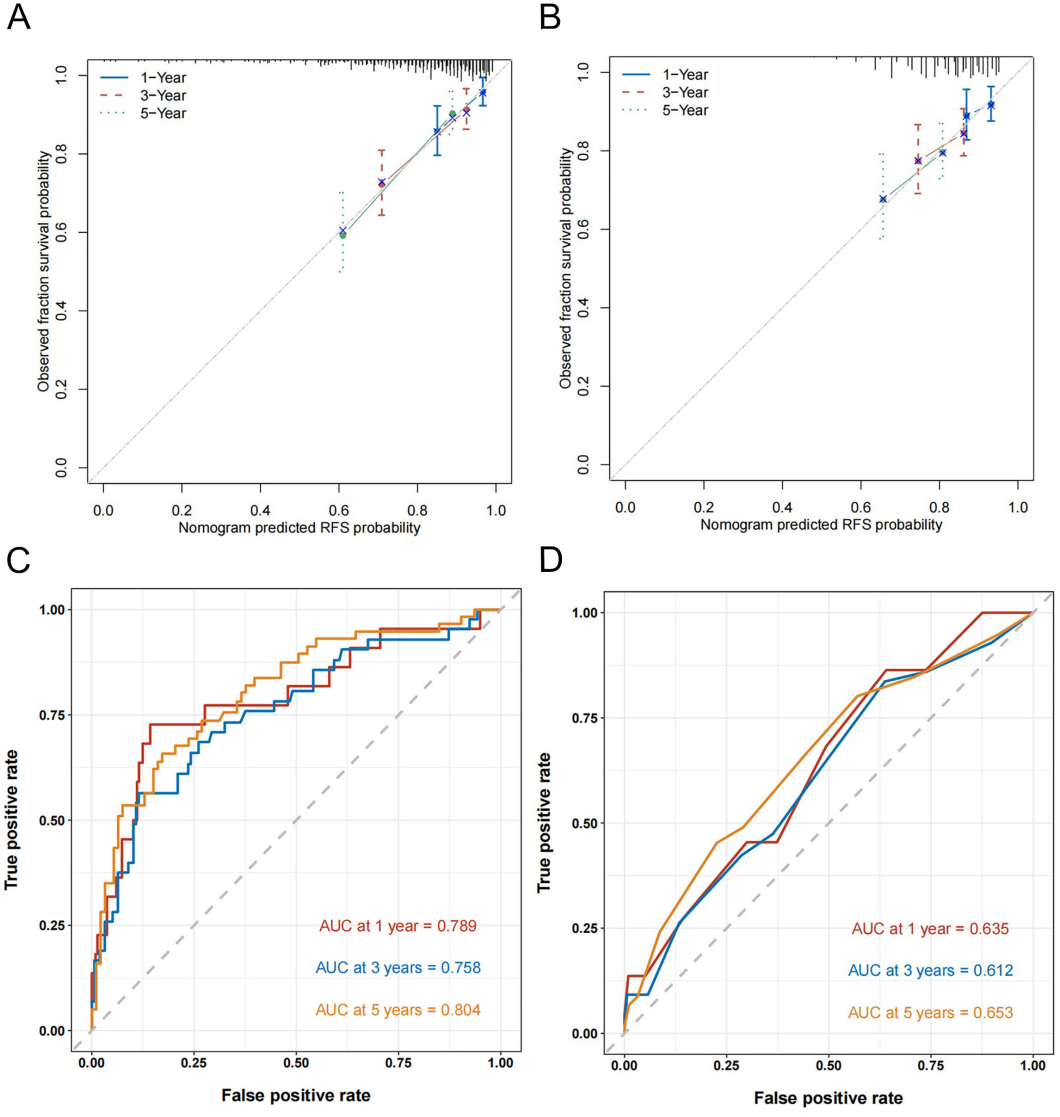
Calibration curves and time-dependent ROC curves for the prediction of 1-, 3- and 5-year RFS. **(A)** Calibration curve of the nomogram. **(B)** Calibration curve of the EORTC recurrence risk score. **(C)** Time-dependent ROC curve of the nomogram. **(D)** Time-dependent ROC curve of the EORTC recurrence risk score. ROC, receiver operating characteristic; RFS, recurrence-free survival; EORTC, European Organization of Research and Treatment of Cancer.

**Figure 6:**
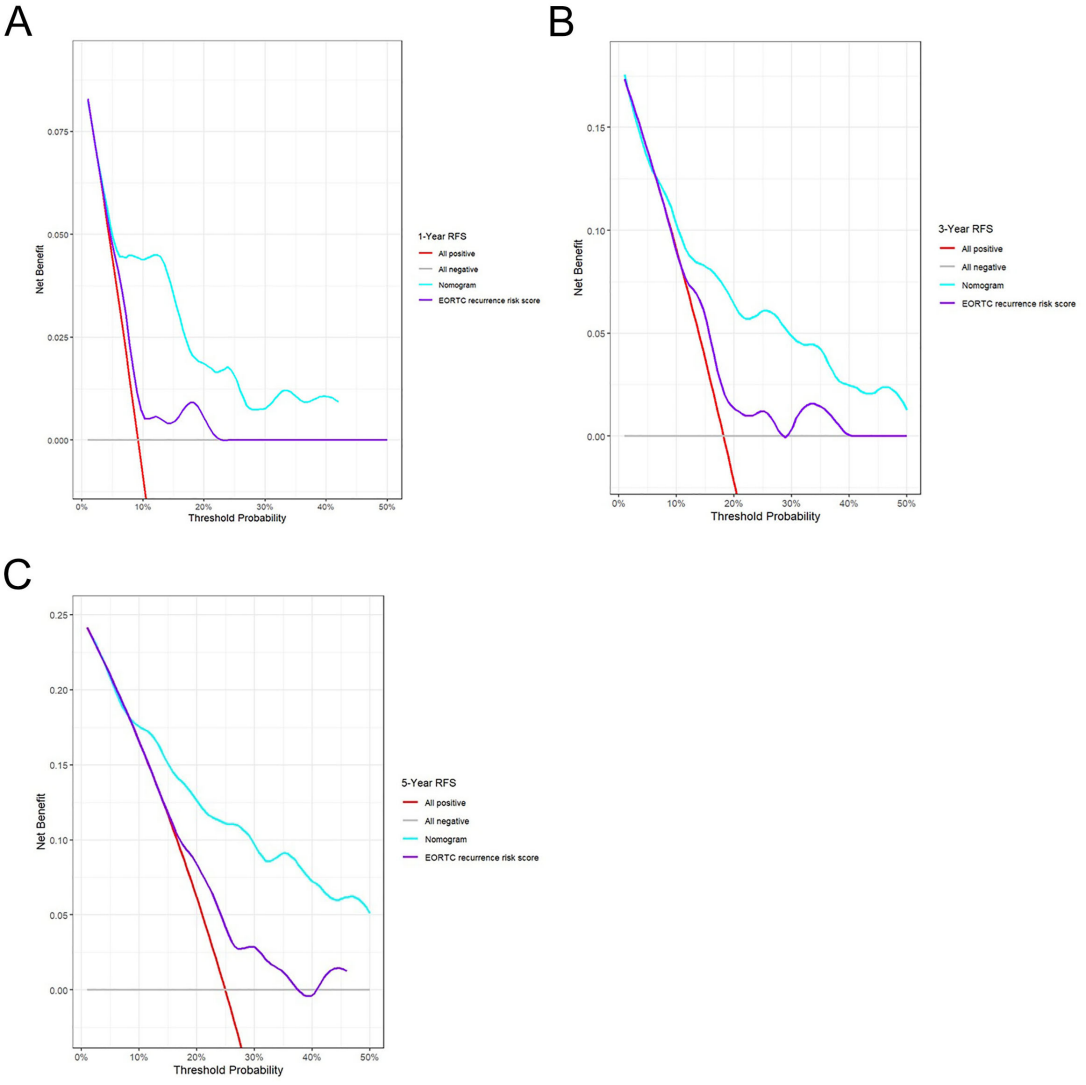
Decision curve analyses of the nomogram. Decision curve analyses for 1-year **(A)**, 3-year **(B)** and 5-year **(C)** RFS prediction. RFS, recurrence-free survival; EORTC, European Organization of Research and Treatment of Cancer.

To develop a tool for predicting 1-, 3-, and 5-year RFS, a nomogram was constructed using the six independent predictors derived from the multivariate Cox analysis: age, history of abdominal surgery, concomitant CIS, tumor size, tumor stage, and RLR (**Figure 4**). The calibration curves generated from 1000 bootstrap resamples demonstrated that, at 1, 3, and 5 years, RFS probabilities predicted by the nomogram (**Figure 5A**) were closer to the ideal model compared to those predicted by the EORTC recurrence risk score model (**Figure 5B**). After internal validation with 1000 bootstrap repetitions, the calibrated C-index of the nomogram was 0.729, indicating favorable discriminative ability. This C-index was higher than that of the nomogram model without RLR (C-index = 0.706) and the EORTC recurrence risk score model (C-index = 0.614) (**Table 4**). Time-dependent ROC analysis revealed that AUC for predicting 1-, 3-, and 5-year RFS by the nomogram were 0.789, 0.758, and 0.804, respectively (**Figure 5C**). These values were all higher than the corresponding AUC of the EORTC recurrence risk score model (1-year: 0.635; 3-year: 0.612; 5-year: 0.650) (**Figure 5D**). At each time point analyzed (1, 3, and 5 years), DCA demonstrated that, across a wide range of threshold probabilities, the application of the nomogram yielded a higher net clinical benefit than both the “treating all patients” and “treating no patients” strategies, as well as the strategy based on the EORTC recurrence risk score (**Figures 6A–C**).

**Table 4:** Comparison of C-indices of different models.

Models	C-index	Optimism	95%CI	Corrected C-index
EORTC recurrence risk score	0.614	<0.001	(0.542, 0.686)	0.614
Nomogram without RLR	0.720	0.014	(0.651, 0.788)	0.706
Nomogram*	0.744	0.031	(0.677, 0.812)	0.729

* Age, history of abdominal surgery, tumor size, tumor stage, concomitant CIS, RLR were included in the nomogram for recurrence-free survival prediction.

CI, confidence interval; EORTC, European Organization of Research and Treatment of Cancer; RLR, red blood cell distribution width-to-lymphocyte ratio; CIS, Carcinoma in situ.

### Subgroup analysis

**Figure 7:**
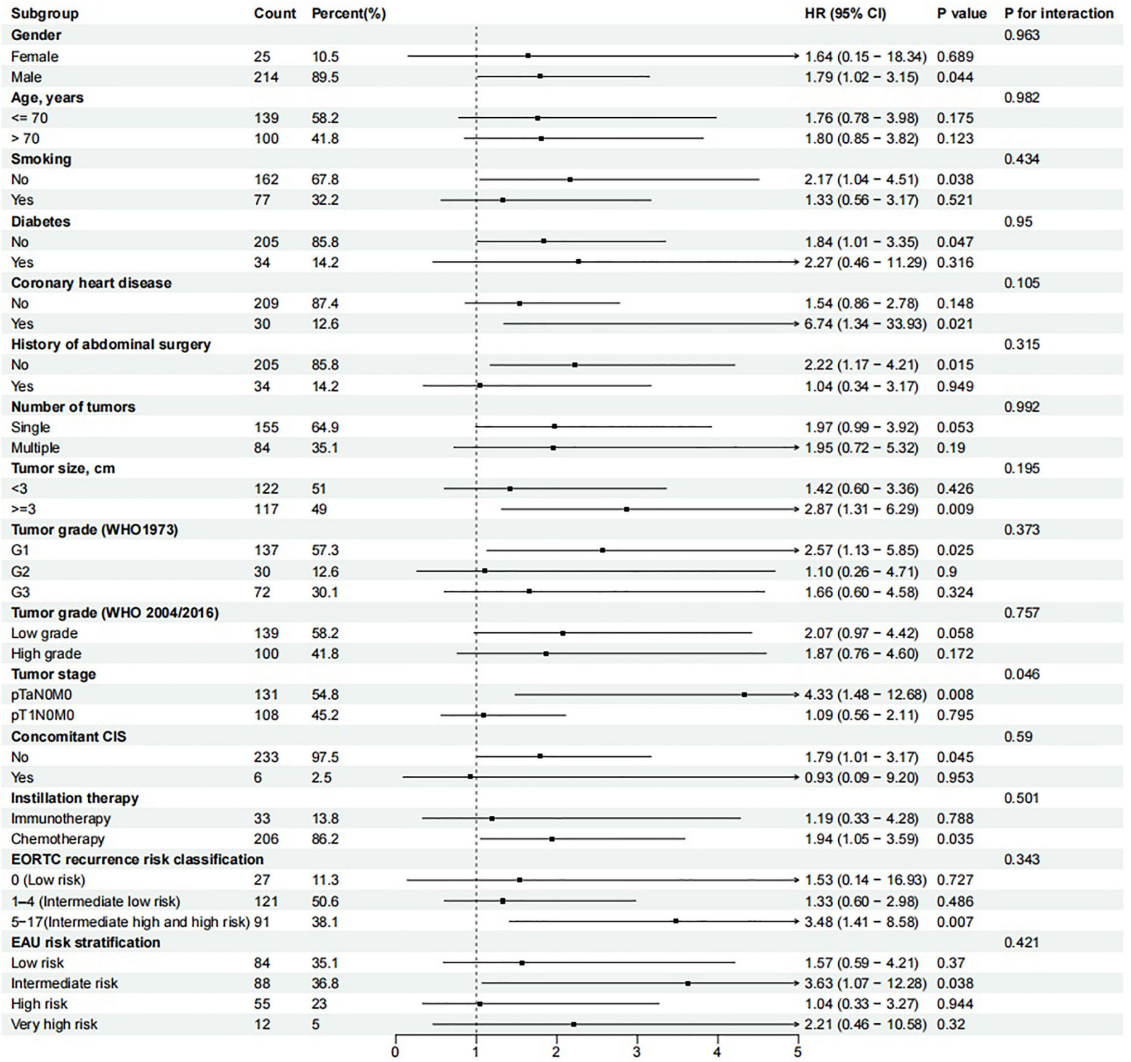
Subgroup analysis of RLR for RFS. CIS, Carcinoma in situ; EORTC, European Organization of Research and Treatment of Cancer; EAU, European Association of Urology.

Subgroup analyses were performed to evaluate potential interaction effects (**Figure 7**). A significant interaction was observed only for tumor stage (P for interaction = 0.046), indicating that the association between an elevated RLR and poorer RFS differed between pTaN0M0 and pT1N0M0 tumors. Specifically, high RLR was a strong and significant predictor of poor RFS in patients with pTaN0M0, but not in those with pT1N0M0. No significant interactions were found for other variables (all P for interaction > 0.05), suggesting a consistent prognostic effect of RLR across these subgroups.

## Discussion

Improving outcomes in NMIBC requires the discovery of validated biomarkers to predict recurrence, which is key to developing individualized treatment approaches. In this study, we investigated the preoperative RLR is a novel and independent factor associated with recurrence risk in NMIBC patients following TURBT. Patients with an elevated RLR exhibited significantly worse RFS and OS according to Kaplan-Meier analysis. These results position RLR as a promising candidate biomarker that warrants further investigation in the prognostic assessment of NMIBC.

To enhance methodological rigor, the optimal cutoff for RLR was determined using time-dependent ROC analysis, as opposed to standard ROC method. Standard ROC curves are suboptimal for survival data, as they ignore both the time-dependent nature of prognostic accuracy and the presence of censored observations (16). The time-dependent ROC method dynamically defines cases and controls at specific follow-up times (e.g., 1 years), providing a cutoff that reflects the marker’s actual discriminatory power for predicting recurrence at those clinically relevant horizons. This approach minimizes the overfitting bias inherent in generating a single, data-driven cutoff from a standard ROC curve, thereby strengthening the validity and clinical applicability of our RLR-based prognostic stratification (17, 18).

Previous studies have established the association of the NLR and PLR with adverse outcomes in NMIBC (19, 20). In the present study, RLR demonstrated a comparative advantage, with higher AUC values for predicting both 1-year and 3-year RFS compared to these traditional markers (NLR and PLR). The underlying mechanisms linking a high RLR to poor prognosis are likely multifactorial, primarily involving the interplay of systemic inflammation, immune surveillance evasion, and the tumor microenvironment. RDW, a standard parameter in a complete blood count, is not only routinely used to diagnose anemia but is also closely associated with chronic inflammation, malnutrition, and unfavorable prognosis in various cancers (21, 22). Conversely, lymphocytopenia indicates an impaired anti-tumor immune response (23). In bladder cancer, chronic inflammation and associated inflammatory markers have been established to be closely linked to tumorigenesis and progression (24–26). A state of chronic inflammation, characterized by the sustained presence of pro-inflammatory cytokines such as IL-6 and TNF-α, can foster a microenvironment conducive to tumor cell proliferation, survival, and angiogenesis (27). Lymphocytes, on the other hand, are central effectors of the anti-tumor immune response; a reduction in their count suggests weakened immune surveillance, thereby diminishing the body’s capacity to eliminate minimal residual disease or circulating tumor cells (28). Therefore, an elevated RLR, reflecting both high RDW (indicative of heightened inflammation) and low lymphocyte count (indicative of immunosuppression), essentially captures a dual pathophysiological state of intensified inflammation and suppressed immunity. This imbalance likely promotes disease recurrence by disrupting the homeostasis of the bladder tumor microenvironment (29), which may underlie the predictive utility of RLR.

Furthermore, our analysis identified a history of abdominal surgery as an independent risk factor for recurrence in our cohort. The mechanisms underlying this association remain speculative but may involve postoperative intra-abdominal adhesions fostering a chronic inflammatory state (30), The mechanisms underlying this association remain speculative but may involve postoperative intra-abdominal adhesions fostering a chronic inflammatory state (31). To translate these findings into a potential clinical tool, we constructed and internally validated a prognostic nomogram incorporating the RLR for predicting RFS. The nomogram showed improved performance compared to the model based solely on the EORTC recurrence risk score, as reflected by higher AUC values at 1, 3, and 5 years (0.789, 0.758, and 0.804 vs. 0.635, 0.612, and 0.650, respectively), a higher overall C-index, better calibration, and greater net benefit on DCA. This suggests that integrating RLR with traditional clinicopathological factors could enhance the accuracy of individualized prognosis, potentially aiding in the identification of high-risk patients who might benefit from more intensive surveillance or adjuvant therapy. For model comparison, we selected the EORTC recurrence risk score (14) as the benchmark. This choice was based on its validation in a patient population most comparable to ours—those receiving post-TURBT intravesical chemotherapy. In contrast, the CUETO model was developed specifically for patients treated with Bacillus Calmette-Guérin immunotherapy (32), and the updated EAU risk stratification is primarily designed to predict disease progression, not recurrence (15). Although RLR was also independently associated with OS, we did not construct a predictive model for OS due to the limited number of events, to avoid model overfitting and ensure robustness. In conclusion, the preoperative RLR is a promising, inexpensive, and readily accessible biomarker that may provide incremental prognostic value for risk stratification in NMIBC.

A significant interaction with tumor stage was observed in the subgroup analysis for RFS. Specifically, a high RLR served as a strong and independent predictor of worse RFS in the pTaN0M0 subgroup, whereas this association was attenuated and became non-significant in the pT1N0M0 subgroup. While this is an interesting and hypothesis-generating observation, it must be interpreted with considerable caution due to the inherent limitations of subgroup analyses in a study of this sample size. A plausible biological explanation may lie in the distinct pathogenic milieus of Ta versus T1 NMIBC. Stage Ta tumors are confined to the mucosa, and their recurrence may be more strongly influenced by a permissive systemic inflammatory and immune milieu, a state potentially reflected by the RLR (33, 34). In contrast, stage T1 tumors, having invaded the lamina propria, possess greater biological aggressiveness and a more complex, localized tumor microenvironment (35).

Our study has several limitations that should be considered when interpreting the results. First, its retrospective, single-center design carries an inherent risk of selection bias and limits the generalizability of our findings. Second,​ the modest cohort size, particularly within subgroups (e.g., by tumor stage), may reduce the statistical power of certain analyses and increase the risk of overfitting the model. Third, the lack of external validation for the nomogram, derived from a retrospective, single-center cohort, precludes any definitive conclusions regarding its clinical applicability. Fourth, the follow-up protocol employed a uniform schedule for all patients rather than adhering to contemporary risk-stratified surveillance guidelines (e.g., from the EAU). This may have introduced detection bias, although it reflects the standardized retrospective data collection from our institution. Fifth, despite adjusting for known confounders in the multivariate analysis, the influence of unmeasured factors, such as detailed comorbidities (e.g., renal or hepatic dysfunction) and histologic subtype, cannot be ruled out. Sixth, the intriguing finding of “history of abdominal surgery” as a risk factor remains highly speculative without mechanistic support or validation. Finally, while the RLR-based nomogram showed improved discrimination, the modest absolute gains in AUC suggest that RLR may serve as a complementary, rather than a definitively superior, biomarker to existing indices. These limitations underscore the preliminary nature of our findings.

## Conclusion

The findings of this study confirm that preoperative RLR remained an independent prognostic factor in NMIBC patients. An elevated RLR was significantly associated with worse RFS and OS. The RLR-based nomogram demonstrated promising discriminatory ability and net clinical benefit compared to a traditional model in internal validation. However, given the study’s inherent limitations—including its retrospective design, modest sample size, and the absence of external validation—these findings must be considered preliminary and hypothesis-generating. As an easily obtainable parameter from routine blood tests, RLR holds potential for improving risk stratification in NMIBC, but its clinical utility requires confirmation in larger, prospective, and multi-center studies. Future research should also focus on elucidating the biological mechanisms underlying this association and integrating RLR with other molecular markers to build more robust predictive models.

## Data Availability

The original contributions presented in the study are included in the article/supplementary material. Further inquiries can be directed to the corresponding author.
