# Supplementary material for: A nomogram based on the red cell distribution width to lymphocyte ratio as a prognostic tool for non-muscle-invasive bladder cancer: a retrospective study
Source: Front Oncol. 2026 Jan 29;16:1728821. doi: 10.3389/fonc.2026.1728821 (PMC12893947; doi:10.3389/fonc.2026.1728821)
Supplement: Supplementary file 1 [file Table1.docx]

**Table S1 Results from 1-year PFS receiver operating characteristic analysis.**

| Variables | Cut-off value | Sensitivity | Specificity | Youden Index |
| --- | --- | --- | --- | --- |
| NLR | 2.05 | 0.68 | 0.60 | 0.29 |
| PLR | 111.95 | 0.59 | 0.62 | 0.21 |
| RLR | 6.85 | 0.68 | 0.71 | 0.39 |

**Abbreviations:** RLR, red blood cell distribution width-to-lymphocyte ratio; NLR, neutrophil-to-lymphocyte ratio; PLR, platelet-to-lymphocyte ratio.
